# Characterizing the ∑3 boundaries in a cold deformed and annealed pure iron

**DOI:** 10.1016/j.dib.2016.11.098

**Published:** 2016-12-06

**Authors:** Weiguo Wang, Song Chen, Wenzhe Chen

**Affiliations:** School of Materials Science and Engineering, Fujian University of Technology, Fuzhou 350118, China

**Keywords:** Pure iron, ∑3 boundary, Grain boundary inter-connection

## Abstract

This article contains the experimental data for the characterization of ∑3 boundaries in a cold deformed and annealed pure iron. Mentioned data are relevant to the research article “ The inter-connections of ∑3 boundaries in pure iron” (Weiguo Wang, Song Chen, Gregory S. Rohrer, Wenzhe Chen, 2017) [1]. The characterization of ∑3 boundaries was performed through an integrated method including electron backscatter diffraction, stereology based five parameter analysis and crystallographic analysis. This method as formulated can determine the inter-connections of any type grain boundaries with fixed misorientation.

**Specifications Table**

**Table:** 

Subject area	*Materials Science, Crystallography, Stereology, Statistics*
More specific subject area	*Microstructure, Interface, Grain boundary*
Type of data	*Figure*
How data was acquired	*FEI Nova nano 450 field emission scanning electron microscope (FE-SEM), Oxford Aztec electron backscatter diffraction (EBSD)*, *Texture-corrected five parameter analysis software which was developed by Carnegie Mellon University (CMU).*
Data format	*Analyzed*
Experimental factors	*Pure iron (99.9 wt.%) was multi-directionally forged (MDF) at room temperature with a true strain of 4, followed by a annealing at 620 °C for 15 min in vacuum.*
Experimental features	*The raw data of grain orientations were acquired by SEM-EBSD. These raw data were processed into orientation imaging microscopy (OIM) on which it is based the orientations of the grain boundary traces were calculated. Then five parameter analysis was used to determine the grain boundary plane orientation (GBPO), and crystallographic analysis was employed to characterize the inter-connections of ∑3 boundaries.*
Data source location	*School of materials science and engineering, Fujian university of technology, Fuzhou 350118, China.*
Data accessibility	*Data are presented in this article.*

**Value of the data**

• The integrated method for the characterization of ∑3 boundaries provides the information of grain boundary inter-connections (GBIC) in terms of statistical significance.
• Compared to the conventionally used parameters such as grain to grain misorientation and GBPO, GBIC is a more appropriate approach, describing more accurately the grain boundary character in grain boundary engineering (GBE) research.
• Further investigations into GBIC for varied types of grain boundaries will be very significant to the GBE research and applications for body-centered cubic (BCC) metals and high stacking fault energy (SFE) face-centered cubic (FCC) metals.
• GBIC and its distributions are structural necessities for the microstructure evolution, and GBIC research will provide some information on grain growth and texture development.

## 1 Data

**Fig. 1:**
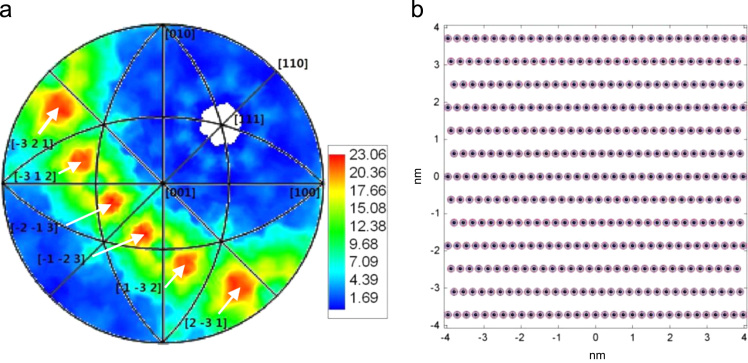
(0 0 1) projections of grain boundary plane orientations (a) and the schematic illustration of atomic configuration of the {1 2 3}/{1 2 3} inter-connections (b) of ∑3 boundaries in a pure iron annealed at 620 °C for 15 min in vacuum after a multi-directional forging with a true strain of 4. (red and blue circles represent the lattice points from the two boundary planes, respectively, and black dots stand for the coincident sites in (b)).

The data reported include information about the inter-connections of ∑3 boundaries in a pure iron (99.9 wt.%) annealed at 620 °C for 15 min in vacuum after a multi-directional forging with a true strain of 4. The inter-connections include (−3 2 1)/(−2 −1 3), (−3 1 2)/(−1 −2 3), (−2 −1 3)/(1 −3 2) and (−1 −2 3)/(2 −3 1) (Fig. 1a).The data also contain information about the schematic illustration of atomic configuration of the {1 2 3}/{1 2 3} inter-connections of ∑3 boundaries (Fig. 1b).

## 2 Experimental design, materials and methods

In order to make a comparison with that of a high purity iron [1], pure iron (99.9 wt.%) was used as experimental material. The sample was subjected to a multi-directional forging at room temperature with a true strain of 4, followed by a annealing at 620 °C for 15 min in vacuum. Then the sample was polished for EBSD measurement.

### 2.1 EBSD measurement

A FEI Nova nano 450 field emission scanning electron microscope (FE-SEM), coupled with an Oxford Aztec electron backscatter diffraction (EBSD) analyzer were used for raw data collection. The EBSD parameters were set to an acceleration voltage of 20 kV, spot size of 4.5, working distance of 9 mm, step size of 2–4 μm and frame size of 1000 μm×800 μm for each mapping. After data collection and data processing, the two-dimensional maps of the orientation imaging microscopy (OIM) were obtained for each of the EBSD mapping and the information of each grain boundary segment was extracted accordingly.

### 2.2 GBPO measurement

A statistical analysis of GBPO concerning the ∑3 boundaries was performed by using the method of texture-corrected five parameter analysis (FPA) [2], [3], [4], [5] which was established based on stereology and statistics. According to stereology, the length fraction of grain boundary segments which were extracted from the OIM was used for representing the area fraction of grain boundary plane in three dimensional grain boundary networks. The GBPO intensity is in the unit of multiples of random distribution (MRD), and it is projected onto (0 0 1) plane.

### 2.3 Determination of GBICs of ∑3 boundaries

Based on the GBPO of ∑3 boundaries with the misorientation of 60°/ [1], the GBICs of this type boundaries was determined based on the following equation [5]

(1) $$\left[\begin{array}{c} h_{2}\\ k_{2}\\ l_{2} \end{array}\right]=\left[\begin{array}{ccc} 2 & -1 & 2\\ 2 & 2 & -1\\ -1 & 2 & 2 \end{array}\right]\left[\begin{array}{c} h_{1}\\ k_{1}\\ l_{1} \end{array}\right]$$

where {h_1_ k_1_ l_1_} and {h_2_ k_2_ l_2_} are the Miller indexes of the inter-connected two planes.
