# Supplementary figures and images for: Characterizing the ∑3 boundaries in a cold deformed and annealed pure iron
Source: Data Brief. 2016 Dec 6;10:294–7. doi: 10.1016/j.dib.2016.11.098 (PMC5156596; doi:10.1016/j.dib.2016.11.098)

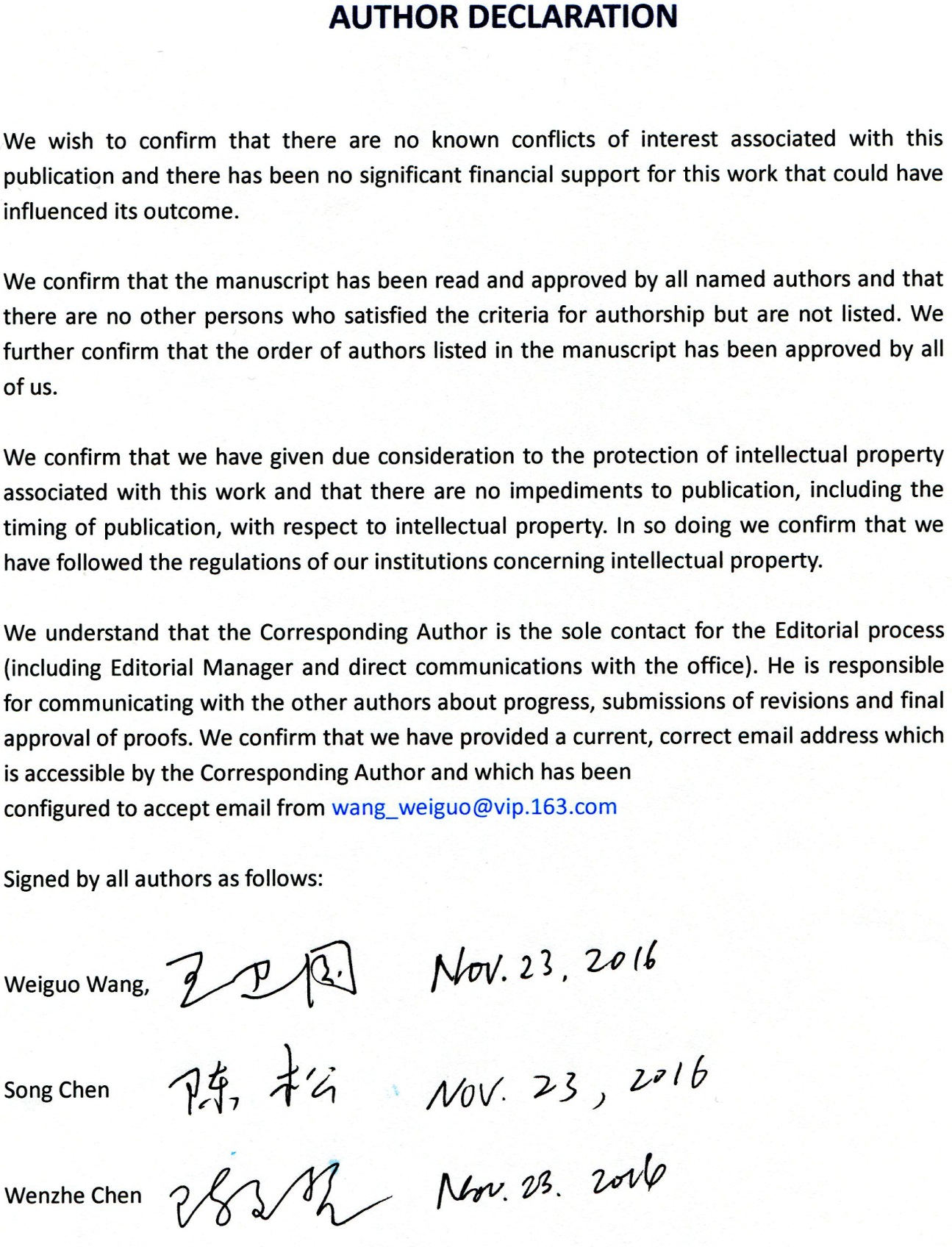

Supplement: Supplementary file 1 — Supplementary material [file mmc1.docx]
